# Candidates’ perceptions of the revised Diploma in HIV Management curriculum and study programme: A cross-sectional survey

**DOI:** 10.4102/jcmsa.v4i1.453

**Published:** 2026-09-11

**Authors:** Madeleine Muller, Oladele V. Adeniyi, Eric Buch

**Affiliations:** 1 Department of Family Medicine & Rural Health, Faculty of Medicine and Health Sciences, Walter Sisulu University, East London, South Africa; 2 School of Health Systems and Public Health, University of Pretoria, Tshwane, South Africa; 3 Colleges of Medicine of South Africa, Johannesburg, South Africa

**Keywords:** colleges of medicine South Africa, college of family physicians, Diploma in HIV Management, candidates’ performance, structured curriculum, study programme, South Africa

## Abstract

**Background:**

The College of Family Physicians (CFP) revised the Diploma in HIV Management (Dip HIV Man) curriculum and, in 2021, introduced a 9-week structured-learning programme with linked resources to assist candidates in preparing for the assessment. This study determines the perceived contributions of the revised curriculum and the structured-learning programme to candidates’ preparation and performance in the Dip HIV Man examination.

**Methods:**

A cross-sectional online survey (Aug–Oct 2023) was sent to all 545 candidates who wrote the Dip HIV Man examination in 2022 and first semester 2023. Likert scale and open-ended data were collected via Google Forms. Descriptive and logistic regression analyses were performed using open statistical software jamovi.

**Results:**

Among 545 candidates, 199 responded (36.5%); most were medical officers working in district or tertiary hospitals with 1 year – 5 years of experience. Most used the revised curriculum and rated resources as relevant and high-quality. Half engaged in the study programme, with high perceived value. Frequent use of curriculum and especially resources seemed associated with passing, with resource use showing the most consistent independent effect. Study group engagement showed weaker, inconsistent associations. Clinical HIV exposure was not associated with success. Overall, 90.4% reported that completing the diploma helped their clinical decision-making.

**Conclusion:**

The candidates found the revised Dip HIV Man curriculum, linked resources and study programme valuable in their examination preparation and perceived it as helpful in their clinical work. However, increased awareness among doctors and tailored resources is needed for further improvements.

**Contribution:**

This study underscores the value of structured educational interventions in postgraduate medical education and provides insights into optimising future examination preparation.

## Introduction

South Africa has an estimated human immunodeficiency virus (HIV) prevalence rate of 13.9%, with approximately 8.45 million people living with HIV in 2022. Nationally, 71.9% of households visit public health facilities for medical care.^1^ More than a third of these have advanced HIV disease at diagnosis and initiation of anti-retroviral therapy (ART), and an increasing number of patients re-engage with care with advanced stages of disease after a period of being lost to follow-up.^2^ The Diploma in HIV Management (Dip HIV Man) is a post-graduate qualification offered by the College of Family Physicians (CFP) under the Colleges of Medicine of South Africa (CMSA).^3^ The Dip HIV Man targets all categories of medical doctors, including specialists involved in the care of people living with HIV. The Dip HIV Man ensures that clinicians in primary and secondary healthcare are capacitated to provide care to a large number of people with HIV in South Africa, including those with advanced HIV disease.

Infectious disease specialists initially supervised and set the Dip HIV Man examination, but in 2019, the CMSA decided that, as the purpose of the diploma was to target primary and secondary care clinicians. The CFP should take over all aspects of the assessment. In line with the CMSA’s move towards competency-based education,^4^ the CFP made several changes to the curriculum structure, assessment and examination preparation guidance. The revised Dip HIV Man was the first CMSA diploma to incorporate professional activities into its design and to include a structured blueprint for assessment. Professional activities (PAs) are units of professional practice that capture essential competencies that trainees must become proficient in before working independently.^5^ It was not appropriate to use the term *entrustable* PAs, as these activities have not been observed within clinical practice (The Dip HIV Man in its current form only tests the application of knowledge through a written paper). Twenty-seven PAs were formulated, subdivided into 84 smaller (observable) activities, and each linked to specific, recommended resources. Examples of PAs include ‘managing patients with HIV with a high creatinine level’ and ‘managing patients on first-line ART with virological failure’.^3^

Professional activities serve as a helpful bridge between the curriculum and learning outcomes, competency frameworks and everyday clinical activities.^6^ An examination blueprint was designed, using each professional activity’s impact factor to determine its weight in the assessment, and the final examination was modified to consist of two high-quality single-best-answer papers. These changes ensured that the assessment covered the entire Dip HIV Man curriculum and was essential to improve the examination’s content validity.^7,8^ The new curriculum was introduced at the first-semester examination in 2020.

As part of the CMSA’s curriculum and assessment overhaul, it also decided to focus on better preparing candidates for its diplomate examinations.^4^ In 2021, a guided learning 9-week study support programme was launched for the Dip HIV Man. The programme was designed around the 27 PAs, and all candidates who had applied for the examination were encouraged to sign up. The 9 weeks included pre-recorded webinars, tools and weekly tips that reinforced the essential aspects of each professional activity. The regulations, curriculum with linked resources and study programme outline were made available on the CMSA website, and candidates who signed up for the programme received weekly emails with the relevant content.

While it was reasonable to assume that the candidates found the revised Dip HIV Man curriculum accessible and useful, it is unclear how much benefit they derived from it. This study, therefore, primarily aimed to describe candidates’ perceptions of the contributions of the revised Dip HIV Man curriculum, linked resources and the study programme to their examination preparation. It hoped to explore associations between candidates’ self-reported engagement and self-reported examination success, and finally, to describe the perceived impact on clinical practice. Findings from the study would help the CMSA evaluate the recent changes to the Dip HIV Man and identify areas for improvement and best practices to be shared with other diploma course designers.

## Research methods and design

### Study design and setting

This cross-sectional analytical survey was conducted between 15 August 2023 and 15 October 2023. An online survey platform was used to collect the data from eligible participants. All doctors who wrote the Dip HIV Man examination in the previous three semesters, from 2022 (two semesters) to 2023 (first semester), were invited to participate in the study.

### Study population and sample size

A total of 545 candidates who wrote the Dip HIV Man in the two semesters of 2022 and the first semester of 2023 were eligible to participate in the study. All 545 candidates were sent an invitation link via email and Short Message Service (SMS). A total of 199 consented and completed the survey. It was not possible to determine how many candidates opened the survey.

### Data collection

Both email and bulk SMS were used to distribute the survey questionnaire and consent form to the eligible candidates on the CMSA database. An anonymous online survey questionnaire designed on Google Forms was used as a data collection tool. Participants received weekly reminders by email and/or bulk SMS. The survey questionnaire was based on the study objectives, using educational evaluation tools to capture the impressions of the candidates.^9,10^ The research team assessed the questionnaire for face and content validity and deemed it adequate to achieve the study objective without additional piloting. The questionnaire is available on request from the corresponding author.

The questionnaire comprised a section on demographic data and a set of Likert scale questions to elicit candidates’ perceptions of the new Dip HIV Man curriculum structure, the value of the recommended, linked resources, and the 9-week guided-learning study support programme. Participants were asked to rate their preparation, use of the resources and study group activities using a five-point Likert scale: 1 (strongly disagree or low) to 5 (strongly agree or high). These scores enabled an analysis of trends and correlations among variables. Finally, participants were asked whether they had passed or failed the Dip HIV Man exam. As surveys were anonymous, pass or fail reports were not linked to CMSA results and therefore not verified.

### Data analysis

A complete dataset was generated on an Excel spreadsheet and used to analyse the demographic data. Data were analysed for possible associations using the free, open-source statistical software jamovi.^11^ Descriptive statistics were used to summarise candidate characteristics and examination outcomes. Categorical variables were reported as frequencies (*n*) and percentages (%). Associations between each academic engagement variable (use of the detailed Dip HIV Man curriculum, use of prescribed resources, engagement with the study group and exposure to HIV patients in clinical practice) and examination success (pass or fail) were examined using binomial logistic regression models. Examination success (pass or fail) was self-reported and not verified against CMSA results (as surveys were anonymous). Covariates were selected a priori based on their theoretical relevance. Responses were mostly complete, except that two of the surveys did not report on examination success (whether they passed or failed). Due to the limited number of candidates who failed the examination (27 failure events), the number of outcome events was insufficient to support a fully adjusted multivariable logistic regression model including all covariates simultaneously, in keeping with recommended events-per-variable considerations for logistic regression. Therefore, for each primary exposure variable, an unadjusted (crude) logistic regression model was first fitted, followed by a series of partially adjusted models in which the exposure was adjusted separately for each of the other engagement variables. This approach was used to explore the robustness of the associations and the potential influence of confounding while minimising overfitting. To further reduce the number of outcome events, a pragmatic approach was used to collapse Likert categories to differentiate between candidates with average to high engagement in comparison with those with little or no engagement. Complete case analysis was used for the descriptive data, while only the 197 cases that reported an examination outcome were used for the logistic regression. Results are presented as odds ratios (ORs) with 95% confidence intervals (CIs) and corresponding *p*-values.

A *p*-value of less than 0.05 was considered statistically significant. The authors verified all analyses and outputs.

#### Research team and management of potential bias

This study formed part of a programme evaluation of the Dip HIV Man and was conducted by the CMSA. The lead author is the convenor of the Dip HIV Man and led the educational team that restructured the Dip HIV Man curriculum and designed and managed the study programme. The third author is in a position of leadership within the CMSA. To minimise bias, the survey was administered after completion of the examination and distribution of results; participation was voluntary; responses were anonymous and non-participation had no influence on examination outcomes or future interactions with the programme. Data analysis was undertaken using de-identified data, and examination results were not available to the researchers.

### Ethical considerations

Ethical approval was obtained from the Human Research Ethics Committee (HREC) of the Faculty of Health Sciences of the University of Stellenbosch (HREC Reference No: N23/06/065). Participants completed an online consent form indicating their voluntary participation in the study. Participation had no impact on diploma examination outcomes, which the CMSA had published before data collection. Survey questionnaires were completed anonymously online without any participant identifiers. The rights to privacy and confidentiality were respected during and after the study in accordance with the Helsinki Declaration and Good Clinical Practice Guidelines.

## Results

### Baseline characteristics

Overall, 199 complete responses were obtained from 545 eligible candidates (36.5% response rate). The majority of participants were medical officers (66.8%), followed by community service doctors (18.1%), registrars (6.5%) and general practitioners (4.5%). Only five were specialists. Most participants worked in district hospitals (26.6%), followed by tertiary hospitals (24.1%), primary healthcare or community health centres (19.1%) and regional hospitals (17.6%). Regarding clinical experience, a substantial majority had 1 year – 5 years’ work experience (*n* = 145, 72.9%). Participants were geographically distributed across all nine provinces, with the highest representation from Gauteng (28.1%), Western Cape (22.1%) and KwaZulu-Natal (16.1%). Most participants reported seeing patients with HIV as part of their broader clinical duties (73.9%), while 15.6% predominantly managed patients with HIV, and 1.5% worked exclusively with patients with HIV. Respondents were evenly distributed across the three semesters; 53 wrote the examination in the first semester of 2022 (26.6%), 77 in the second semester of 2022 (38.7%), and 69 in the first semester of 2023 (34.7%) (Table 1). Of the 199 candidates, 197 reported on whether they passed the Dip HIV Man examination. The pass rate for the 545 candidates across the three examinations was 78.4%, while the pass rate for the 197 candidates in the study sample was 86.3%.

**TABLE 1:** Demographic characteristics of the respondents.

Variables	Frequency (*n*)	%
**Job title**		
Community service officer	36	18.1
Medical officer	133	66.8
General practitioner	9	4.5
Registrar	13	6.5
Specialist†	5	2.5
Research medical doctor	1	0.5
Unemployed	2	1.0
**Type of facility**		
Primary Healthcare or Community Health Centre	38	19.1
District hospital	53	26.6
Regional hospital	35	17.6
Tertiary hospital‡	48	24.1
Private practice	10	5.0
Non-Governmental Organisation	4	2.0
Academic or Research	6	3.0
Other	5	2.5
**Years of experience as a doctor (Years)**		
≤ 1	4	2.0
1–5	145	72.9
6–10	37	18.6
≥ 10	13	6.5
**Province**		
Western Cape	44	22.1
Eastern Cape	21	10.6
Northern Cape	6	3.0
KwaZulu-Natal	32	16.1
Free State	7	3.5
Gauteng	56	28.1
Limpopo	7	3.5
Mpumalanga	9	4.5
North West	13	6.5
Overseas	2	1.0
Unemployed	2	1.0
**Exposure to patients with HIV in clinical practice**		
Exclusively patients with HIV	3	1.5
Predominantly patients with HIV	31	15.6
HIV in the wider scope of patients seen	147	73.9
I do not see any patients with HIV	4	2.0
Not in clinical practice	14	7.0
**Timing of writing the Dip HIV Man examination**		
First semester (2022)	53	26.6
Second semester (2022)	77	38.7
First semester (2023)	69	34.7

HIV, human immunodeficiency virus.

† , Specialist: Physicians (*n* = 3); Paediatrician (*n* = 1); Research scientist (*n* = 1);

‡ , Tertiary Hospital Department: Internal Medicine (*n* = 25); Family Medicine (*n* = 1); Paediatrics (*n* = 9), Psychiatry (*n* = 1); Emergency (*n* = 4); Virology (*n* = 3); Dermatology (*n* = 1); Research (*n* = 2); Obst. & Gynae (*n* = 1).

### Perceptions about the usefulness of the revised Diploma in HIV Management curriculum

Of the 199 candidates, 175 (87.9%) reported average, more than average, and high use of the revised Dip HIV Man curriculum structure for their preparation, with almost two-thirds (*n* = 129; 64.8%) confirming they used it often. Three-quarters of the candidates (*n* = 150; 75.4%) reported that the curriculum was clear about what they needed to prepare for the examination (Table 2).

**TABLE 2:** Engagement with the revised Diploma in HIV Management curriculum.

Variables	Frequency (*n*)	%
**The Dip HIV Man curriculum was clear about what I needed to prepare for the examination**		
Strongly agree	65	32.7
Agree	85	42.7
Neutral	34	17.1
Disagree	13	6.5
Strongly disagree	2	1.0
**How often did you use the curriculum and blueprint to guide your preparation for the examination?**		
Highly used	84	42.2
More than average use	45	22.6
Average use	46	23.1
Rarely used	16	8.0
Never	8	4.0

Dip HIV Man, Diploma in HIV Management.

### Perceptions of the recommended resources for the Diploma in HIV Management

A substantial majority of respondents (*n* = 167, 84%) agreed or strongly agreed that the resources linked to each activity in the curriculum were relevant, and 83.5% (*n* = 166) agreed or strongly agreed that the resources were of good quality. The majority of respondents felt that they were able to work through most of the resources (*n* = 152, 81.4%), while 18.6% found it impossible to cover all the material. Similarly, the majority of respondents (*n* = 127, 62.3%) felt that the volume of resources provided was just right, while 29.1% (*N* = 58) felt there were too many and 8.5% (*n* = 17) felt there were not enough resources (Table 3).

**TABLE 3:** Candidates’ perceptions of the Diploma in HIV Management resources.

Variables	Frequency (*n*)	%
**I found the resources were relevant**		
Strongly agree	67	33.7
Agree	100	50.3
Neutral	28	14.1
Disagree	3	1.5
Strongly disagree	1	0.5
**The resources were of good quality**		
Strongly agree	60	30.2
Agree	106	53.3
Neutral	29	14.6
Disagree	3	1.5
Strongly disagree	1	0.5
**Ability to study all the resources**		
Impossible to work through all the resources	37	18.6
I was able to work through *most* of the resources	123	61.8
I was able to work through *all* the resources	23	11.6
I was able to work though the resources more than once or several times	16	8.0
**I feel the volume of resources was appropriate**		
Not enough	17	8.5
Just right	124	62.3
Too many	58	29.1

### Perceptions of the Diploma in HIV Management study programme

Half the respondents (50.3%) had joined for either part of or the whole study programme, and an additional 24.1% (*n* = 48) had downloaded the material and studied it independently. The remaining 51 candidates had not participated in the 9-week study programme. Of the 148 respondents who engaged with the programme, 87.2% (*n* = 129) actively participated in the weekly tasks, while 12.8% (*n* = 19) participated only occasionally or not at all. A substantial majority (86.5%) agreed or strongly agreed that the study programme was valuable in their preparations for the examination (Table 4).

**TABLE 4:** Respondents’ experience of the Diploma in HIV Management 9-week study programme.

Variables	Frequency (*n*)	%
**Access to 9-week study programme**		
None	28	14.1
Looked at it but did not use it	23	11.6
Downloaded the study material but did not join the weekly study programme	48	24.1
Joined part of the study programme	26	13.1
Joined all of the study programme	74	37.2
**Engagement with the study programme of candidates who accessed it (*n* = 148)**		
Every week	67	45,3
More than half the time	41	27.7
About half the time	21	14.2
Occasionally or not at all	19	12.8
**I found the 9 week-study programme and/or material useful in preparing for the exam (*n* = 148)**		
Strongly agree	99	66.9
Agree	29	19.6
Neutral	16	10.8
Disagree or strongly disagree	4	2.7

### Association between use of the revised Diploma in HIV Management curriculum, resources and study group and success

A fully adjusted multivariable logistic regression model was not possible due to the small number of outcome events. Separate bivariate adjustments for specific exposures were tested to identify possible associations between the use of the curriculum, resources, study programme and extent of exposure to HIV in the workplace and success in the examination. A detailed table with all results is available on request from the corresponding author. Only a small number of potential associations were identified, and wide confidence intervals in some indicate a lack of precision.

#### Use of Diploma in HIV Management curriculum

Candidates who frequently used the detailed Dip HIV Man curriculum may have a higher odds of examination success. In contrast, average use of the curriculum was not significantly associated with examination success in either unadjusted or adjusted models (Table 5). In the unadjusted analysis, there was an indication that candidates who reported often or high use of the curriculum had higher odds of passing compared to those who never used it (OR 7.50, 95% CI 2.47–22.76; *p* < 0.001). This association remained statistically significant after adjustment for engagement with the study group (OR 5.71, 95% CI 1.77–18.42; *p* = 0.004) and for the level of clinical HIV exposure in their workplace (OR 7.67, 95% CI 2.51–23.48; *p* < 0.001), suggesting an independent association between frequent curriculum use and examination success in these models. However, after adjustment for the extent to which the candidate used the prescribed resources, the association was attenuated and no longer statistically significant (OR 2.85, 95% CI 0.74–10.98; *p* = 0.128), indicating that the benefit of using the structured curriculum is operationalised through the use of resources.

**TABLE 5:** Logistic regression analysis: Possible associations between engagement with the revised Diploma in HIV Management curriculum, resources and study group and successful outcome in the Diploma in HIV Management examination.

Exposure	Comparison	Unadjusted OR	*p*-value	Adjusted	Adjusted OR	*p*-value
Curriculum: Often or high use	Curriculum: Never Used	7.50 (2.47–22.76)	< 0.001	Adjusted for Clinical HIV Exposure in the workplace	7.67 (2.51–23.48)	< 0.001
Curriculum: Often or high use	Curriculum: Never Used	7.50 (2.47–22.76)	< 0.001	Adjusted for Resource Use	2.85 (0.74–10.98)	0.128
Resources: Often or high	Resources: Never or rarely used	13.7 (3.39–55.33)	< 0.001	Adjusted for curriculum use	7.2 (1.44–36.56)	0.016
Resources: Often or high	Resources: Never or rarely used	13.7 (3.39–55.33)	< 0.001	Adjusted for Study group	10.1 (2.24–45.6)	0.003
Resources: Often or high	Resources: Never or rarely used	13.7 (3.39–55.33)	< 0.001	Adjusted for Clinical HIV Exposure in the workplace	14.28 (3.47–58.78)	< 0.001
Study Group: Regular engagement	None or occasional engagement	2.93 (1.06–8.10)	0.038	Adjusted for Clinical HIV Exposure in the workplace	3.0 (1.08–8.39)	0.036

Note: Use of recommended Dip HIV Man Resources.

UOR, unadjusted odds ratio; OR, odds ratio; CI, confidence interval.

Candidates who reported frequent use of the specific resources recommended in the curriculum showed an exploratory association with examination success across all models (Table 5), while average resource use was not significantly associated with passing. In the unadjusted analysis, candidates who reported frequent or high use of resources had odds of passing than those who rarely or never used them (OR 13.7, 95% CI 3.39–55.33; *p* < 0.001), with a very wide confidence interval. This association remained statistically significant after adjustment for the extent of using the recommended curriculum (OR 7.2, 95% CI 1.44–36.56; *p* = 0.016), engagement with the study group (OR 10.1, 95% CI 2.24–45.6; *p* = 0.003) and exposure to HIV patients in clinical environments (OR 14.28, 95% CI 3.47–58.78; *p* < 0.001), indicating a possible independent relationship between frequent resource use and examination success. The wide confidence intervals reflect imprecision due to the small number of failure events, but the size and consistency of the effect suggest that high engagement with the recommended resources may be an important predictor of whether a candidate passes or fails.

#### Engagement with the Diploma in HIV Management study group

Engagement with the study group was associated with examination success in unadjusted analyses, though this relationship was less consistent after adjustment for other academic engagement variables (Table 5). Candidates who reported average engagement with the study group had higher odds of passing compared to those with none or only occasional engagement (OR 3.35, 95% CI 1.14–9.84; *p* = 0.028), and a similar association was observed for those with regular engagement (OR 2.93, 95% CI 1.06–8.10; *p* = 0.038). After adjustment for time spent using the recommended resources or for the extent of curriculum use, these associations were attenuated and were no longer statistically significant, suggesting that the apparent effect of study group engagement may be partly mediated through other forms of academic engagement.

In contrast, after adjustment for the amount of exposure to patients with HIV in clinical practice, both average (OR 3.39, 95% CI 1.15–10.02; *p* = 0.027) and regular engagement (OR 3.0, 95% CI 1.08–8.39; *p* = 0.036) showed an association with passing the examination.

#### Exposure to human immunodeficiency virus patients in clinical practice

A final set of models examined whether the amount of exposure to HIV patients in clinical practice was independently associated with passing the examination (Table 5). There was no evidence of an association between degree of HIV exposure and examination outcome in unadjusted or adjusted models, even after controlling separately for curriculum use, resource use and study group engagement (all ORs close to 1 with wide confidence intervals that included 1; all *p*-values > 0.6).

#### Overall interpretation

Taken together, these analyses suggest that frequent use of the prescribed and recommended resources seems to indicate an association with examination success. The use of the structured curriculum may be important, inasmuch as it guides candidates in using the appropriate resources. Engagement with the study group appears to contribute to success in unadjusted models, but this association is attenuated when resource and curriculum use are taken into account, whereas self-reported exposure to HIV in clinical practice does not seem to have an independent effect on examination outcome.

### Perceived contribution of the Diploma in HIV Management to individuals’ clinical practice

A substantial majority (90.4%) of candidates agreed or strongly agreed that the Dip HIV Man had helped their decision-making in managing patients with HIV. However, a few respondents disagreed or were unsure about the benefit to their clinical practice (9.6%). A substantial majority (93%) agreed or strongly agreed that the section on adult initiation and management of ART had a significant impact on their clinical practice. A similar trend was reported for the section on managing opportunistic infections (91%), adverse effects of ART (93%) and prevention of HIV (90%). The section on management of HIV in children (85%), prevention of vertical transmission and sexual health (86%) and management of HIV with co-morbid diseases (87%) were all deemed very useful. However, the section on managing key populations and adherence support had a slightly lower impact on their clinical practice (81%) (Figure 1).

**FIGURE 1:**
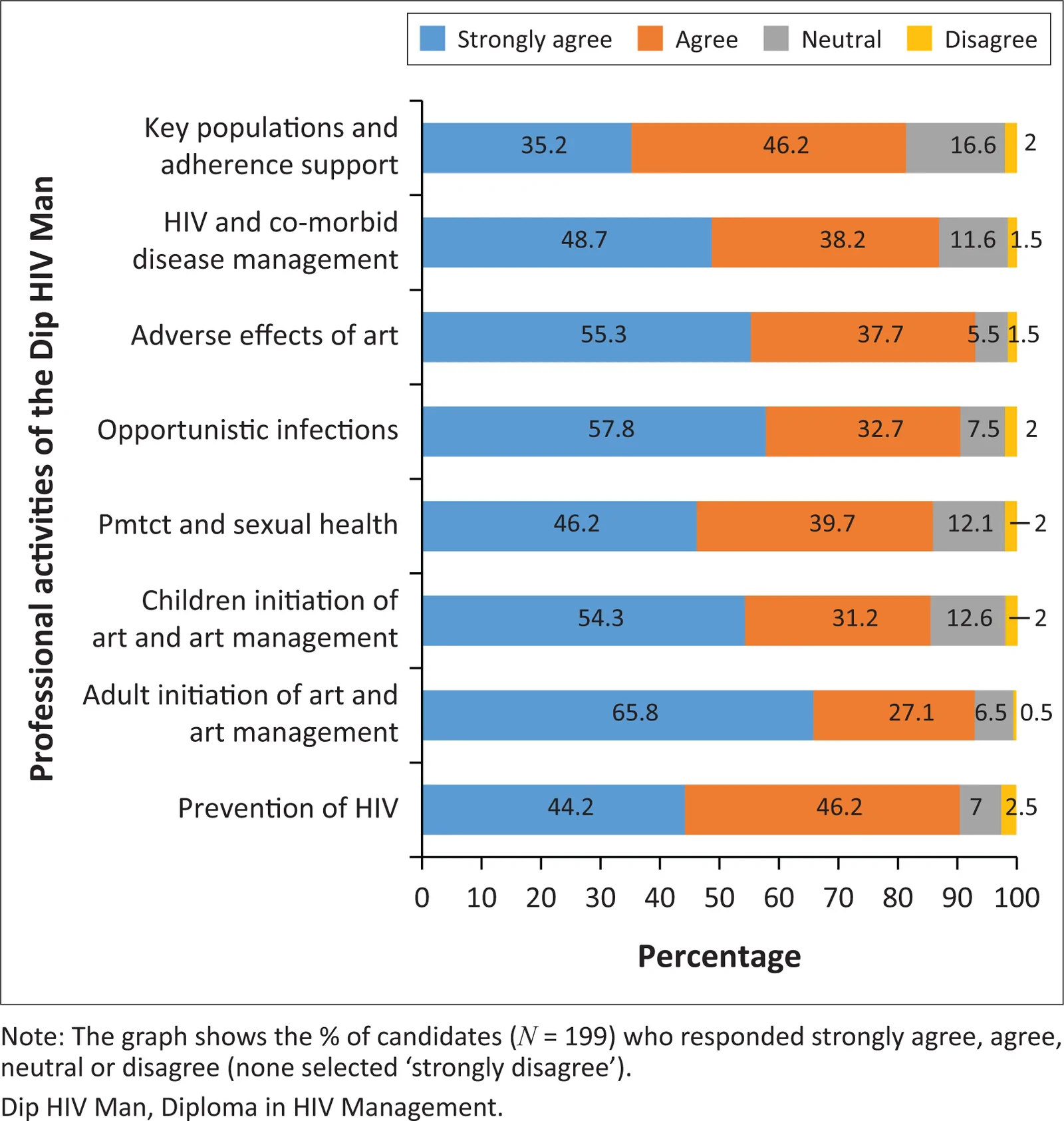
Candidate’s response to the question: ‘This part of the curriculum had a significant impact on my clinical practice’.

## Discussion

The use of structured curriculum and guided study programme in the Colleges of Medicine of South Africa is a new concept recently introduced by the College of Family Medicine.^3^ Whether the candidates for the examination find the curriculum, resources and structured study programme useful in their preparation for the Dip HIV Man examination is unclear. This study addressed an identified knowledge gap by determining the perceived contributions of the revised curriculum to candidates’ preparation and performance in the Dip HIV Man examination. It is expected that lessons learned from one college diploma can be transferred to others. Therefore, the findings from this study will provide feedback to the convenors of the Dip HIV Man and other relevant stakeholders on areas for consolidation and improvement.

Of interest is the geographical spread of doctors across the country and the professional categories, ranging from medical interns to general practitioners and specialists. Participants worked across the entire range of health service delivery in the country, including primary healthcare centres, public tertiary hospitals and private hospitals. It is reassuring to know that doctors across all specialities are committed to ongoing learning in their clinical practice. This aligns with the Health Professional Council of South Africa (HPCSA) stipulation of 30 Continuing Education Unit (CEU) points per cycle.^12^ Patients and society are more assured of healthcare safety when doctors regularly update their knowledge and practice.

The study reports a high level of active engagement with the Dip HIV Man Blueprint and Curriculum, with candidates confirming that it provided sufficient guidance for examination preparation. Every postgraduate programme should have a clear blueprint with a well-defined scope of what is expected from the candidates^13^ and defined resources accessible to the candidates before the examination.^3^ This study highlights how the revised Dip HIV Man curriculum, with its clear, well-defined PAs, helped candidates prepare for the examination. The vast majority of candidates confirmed that the resources were relevant to the examination and were of good quality. Importantly, the candidates reported ease of use of these resources. The study suggests that self-reported engagement with the revised curriculum, particularly the recommended resources, may be important to successful outcomes in the Dip HIV Man examination, although this association may be due to untested confounders such as motivation, prior academic ability and response bias. The structured curriculum acts as a guide, but its benefit depends on the use of the recommended resources. Unsurprisingly, students who engage with detailed information about the level of knowledge and skills (PAs) expected of them and are given specific resources to help them achieve them may be more likely to pass.

Since the coronavirus disease 2019 (COVID-19) pandemic, there has been an exponential increase in online learning platforms, with studies showing high acceptability among undergraduate and postgraduate students.^14,15,16^ At present, the Dip HIV Man online learning platform represents a repository of information. This can be enhanced by introducing interactive, case-based learning, as students value interactive and personalised features.^14^ Introducing online learning into the diplomas has enormous potential to increase reach, engagement and participation.^16^ That said, online learning cannot replace clinical learning and social interactions,^15^ and it will be important to consider how to link it with real-life clinical exposure in the future.

Although those who participated found the distance learning study programme important and immensely helpful in preparing for the Dip HIV Man, there were varying levels of participation. Only half of the respondents in this study engaged actively in the online sessions, with another 24% downloading and studying the resources asynchronously. Participation was voluntary, and not all participants may have been aware of the programme. Enrolling all candidates automatically in the programme will ensure they have access. Challenges with technology and the study platform will need to be addressed.^16^ Given doctors’ diverse schedules and working hours, multiple online programmes targeting different categories of doctors could be explored.

The study has shown that beyond helping candidates pass the examination, the revised curriculum was perceived to impart useful knowledge for their clinical practice. This is aligned with the HPCSA’s requirement for continuing professional development of doctors.^12^ It is also worth noting that candidates found some PAs outlined in the curriculum more useful than others. For instance, the sections on adult initiation and management of antiretroviral therapy and opportunistic infections were the highest-ranked PAs by candidates, followed by the adverse effects of antiretroviral therapy and HIV prevention. While all PAs had to be completed, it is not surprising that each doctor took from the programme what was most useful to their practice. The Dip HIV Man examination, however, is structured so that all PAs are sampled, ensuring that candidates are assessed on all aspects of the curriculum and thereby improving its validity and trustworthiness.^8^

This study confirms the benefit of providing candidates with a clearly structured curriculum, prescribed resources and easy access to study materials to assist in their preparation for the Dip HIV Man Examination. The Dip HIV Man curriculum and study programme improvements are worth emulating across all disciplines in the CMSA.

### Strengths and limitations

This is the first study to elicit examination candidates’ perceptions of the value of the CMSA Dip HIV Man. Online learning platforms offer new opportunities to enhance postgraduate education in sub-Saharan Africa and are affordable, accessible and acceptable to clinicians.^17,18,19^ The convenors of the other CMSA diplomas should consider implementing online academic programmes to improve their examination success rates.

A limitation of this study should be noted: Only 36.5% of the eligible candidates participated in it. Future studies should aim to recruit participants shortly after the examination to increase response rates. The small number of candidates who failed the examination in the study sample limited the analysis of the association of some covariates with examination success, such as years of experience and job title. Examination outcomes (pass or fail) were self-reported and not verified, and may not be a true reflection of the pass rate in this group. The study focused on exploring the candidates’ perceptions rather than on identifying how their engagement with the revised material affects their examination success. Due to the small number of failures in this group, a fully adjusted multiple regression could not be performed to probe for associations. Using separate bivariate adjustments indicated possible associations that may be promising, but wide confidence intervals suggest high imprecision and would require confirmation in prospective studies.

## Conclusion

Candidates for the Dip HIV Man found the revised curriculum, resources and structured study programme valuable in their preparation and in their successful outcomes in the examination. Findings show that active engagement with the curriculum and resources may be crucial to success in the Dip HIV Man examination. Beyond passing the examination, the candidates believed that writing the Dip HIV Man enhanced their clinical competence across various facets of HIV care. However, gaps exist in maximising doctors’ attendance in the 9-week online programme. The findings could be useful for other diploma programmes under the auspices of the College of Medicine of South Africa, which could consider applying this innovative strategy to improve the throughput of their candidates. Further research is needed to measure the impact of writing the Dip HIV Man on the clinical outcomes of patients.
